# Cytomegalovirus Esophagitis in an Immunocompromised Patient

**DOI:** 10.7759/cureus.45634

**Published:** 2023-09-20

**Authors:** Adya A Ali, Sheela Anasseri, Jaafar Abou-Ghaida, Leslie Walker, Tye Barber

**Affiliations:** 1 Osteopathic Medicine, Nova Southeastern University Dr. Kiran C. Patel College of Osteopathic Medicine, Fort Lauderdale, USA; 2 Family Medicine, Broward Health Medical Center, Fort Lauderdale, USA; 3 Family Medicine, Broward Health Medical Center, Fort Lauderdale , USA

**Keywords:** cytomegalovirus, esophagitis, end-organ disease, opportunistic infection, aids, hiv, rectal cancer, squamous cell cancer

## Abstract

Cytomegalovirus (CMV) can present with end-organ disease (EOD), particularly in patients with a CD4 cell count <50/mm^3^. While EOD in immunocompromised patients commonly presents as CMV retinitis (30%) and CMV colitis (5-10%), CMV esophagitis is rare. CMV is the third most common infectious esophagitis following Candida and Herpes Simplex. CMV esophagitis presents with odynophagia, dysphagia, and abdominal pain. Endoscopic exam may reveal large, linear distal esophageal ulcers. Histopathology or serology studies are diagnostic, though serology may be unreliable in the extremely immunosuppressed. Current treatment consists of antivirals such as ganciclovir and valganciclovir. Esophageal disease due to CMV carries a poor prognosis in the immunocompromised. We present the case of a 56-year-old male with a medical history of HIV/AIDS and stage III rectal squamous cell cancer who presented with shortness of breath, weakness, and chronic diarrhea. His HIV was previously well-controlled on antiretroviral therapy. However, due to his malignancy, he was undergoing treatment with chemotherapy and radiation. Initial labs revealed a CD4 count of 42. His clinical course consisted of *Escherichia coli* septicemia, new-onset atrial fibrillation with a rapid ventricular response, worsening pneumonia, possible metastasis, progressive diarrhea, and potential oropharyngeal candidiasis. Despite several broad-spectrum antimicrobial regimens, he remained symptomatic with new complaints of dysphagia and odynophagia. Eventually, the appearance of vesicular lesions on the lips and a repeat CD4 count of 13 garnered a suspicion of HSV or CMV. This complicated case highlights the necessity for a high index of suspicion of rare manifestations of CMV EOD in an immunocompromised patient presenting with confounding clinical symptoms and extensive diagnoses.

## Introduction

Cytomegalovirus (CMV) is an opportunistic double-stranded DNA virus in the *Herpesviridae* family. CMV is spread through contact with contaminated surfaces, transplacentally, during blood transfusions, or through infected bodily fluids such as during the perinatal period or sexual contact [1]. CMV is highly prevalent worldwide, with 40-100 percent of the population estimated to have been exposed to the virus at some point in their lifetime [2].

Once infected, CMV remains latent in mononuclear cells for the lifetime of the individual. Inciting events predisposing patients to an immunocompromised state can lead to reactivation of the virus [1,3]. Usually, the infection is asymptomatic in immunocompetent hosts or may present as a self-limiting, mild mononucleosis-like sickness. Immunocompromised individuals such as patients with acquired immunodeficiency syndrome (AIDS), transplant recipients receiving immunosuppressants, patients with an altered microbiome secondary to antimicrobial use, and patients receiving chemotherapy are not only at a higher risk of illness due to reactivation or initial infection but are also more likely to experience severe manifestations and end-organ disease (EOD) [1]. Patients are at the highest risk for opportunistic infection when the CD4+ T lymphocyte cell (CD4) count is <50 cells/mm^3^ [3].

When CMV is diagnosed, it is usually in people living with HIV/AIDs who are not maintained on a stable antiretroviral therapy (ART) regimen [4]. CMV may affect the retina, lungs, liver, gastrointestinal (GI) tract, and brain. However, the two most common EOD clinical manifestations in AIDS patients are CMV retinitis (30%) and CMV colitis (5-10%) [3,5]. CMV esophagitis, pneumonia, and hepatitis, on the other hand, are rarer manifestations [6]. 

CMV is the third most common cause of infectious esophagitis following Candida and Herpes Simplex Virus [7]. CMV esophagitis can present with odynophagia, dysphagia, and abdominal pain. Endoscopic exam of the GI tract may show large, linear ulcers located in the middle to distal esophagus. The gold standard for detecting CMV is histopathological identification of CMV inclusion bodies [1,8]. Active disease can also be detected by serology studies. However, extremely immunosuppressed patients may have unreliable serology studies and may require further work-up to diagnose a new or reactivated infection [1,9]. Additionally, the widespread availability and use of effective ART has made the incidence of CMV infections and EOD decrease significantly [3,4].

While treatment is generally supportive in immunocompetent patients, severe CMV manifestations are managed with antivirals such as ganciclovir or valganciclovir immediately upon suspicion of a CMV infection [1,3,8]. CMV infections in immunocompetent patients are rarely associated with mortality and generally have a good prognosis. However, in immunocompromised patients, especially those with significant comorbidities, CMV may be related to significant morbidity and mortality [1]. In this case report, we describe a clinically complicated patient with advanced HIV, immunosuppression following chemotherapy treatment for cancer, and septic bacteremia, who was managed and treated for a rare end-organ manifestation of CMV disease.

## Case presentation

We present a 56-year-old male with a medical history of AIDS, stage III p16+ rectal squamous cell cancer, recurrent pneumonia and candidal infections, ADHD, hypertension, benign prostatic hyperplasia, and GERD who presented to the emergency department (ED) with complaints of shortness of breath, weakness, nausea, and chronic diarrhea. He also complained of severe generalized pain and intractable hiccups. He was diagnosed with HIV over five years ago and had a stable ART regimen. He was further diagnosed with rectal squamous cell cancer seven months prior to this hospital admission. He completed 32 sessions of chemotherapy with mitomycin and 5-fluorouracil, with the final session seven days prior to arrival. Radiation treatment was set to conclude in three weeks. Upon completing another session of radiation that morning, the patient described worsening shortness of breath and profuse, uncontrollable diarrhea. The patient had a history of intravenous (IV) drug and tobacco use which he quit 10 years ago. According to the patient, he was adherent to several medications: bictegravir/emtricitabine/tenofovir alafenamide, atomoxetine, propranolol, amiodarone, atorvastatin, finasteride, metoclopramide, bupropion, and baclofen. In the ED, the patient’s respiratory rate was 22 breaths/minute while all other vital signs were within normal limits. 

On physical examination, the patient was moderately anxious with pale upper and lower extremities and macroglossia with a Mallampati score of four, limiting full evaluation of possible thrush. Cardiovascular exam was unremarkable and pulmonary exam was difficult to obtain due to the patient’s restlessness and labored breathing. Initial laboratory findings were significant for pancytopenia, acute kidney injury, hyponatremia, thrombocytopenia (Table 1), and a CD4+ count of 42. The patient was admitted to our team’s care and remained in the hospital for one month prior to discharge with outpatient follow-up. 

**Table 1:** Pertinent laboratory values at presentation *Critical low. ^Found to be 13 cells/mcL 13 days into admission.

Laboratory Value	Normal Range	At Presentation
White cell count (x 10^3^/μL)	4.0 – 11.0	0.31*
Hemoglobin (g/dL)	13.0 - 17.3	9.8
Hematocrit (%)	40 - 50	29.9
Red blood cells (x 10^6^/μL)	4.3-5.8	3.47
Platelet count (x 10^3^/μL)	152 - 324	50
Serum sodium (mEq/L)	135-145	129
Serum potassium (mEq/L)	3.4-5.3	3.8
Blood urea nitrogen (mg/dL)	8-21	23
Creatinine (mg/dL)	0.75-1.2	1.8
Glomerular filtration rate (mL/min/1.73m^2^)	>60	41
CD4 T lymphocyte count (cells/mcL)	430-1800	42^

On the second day of admission, our patient was found to have a fever of 101.3 °F and complained of severe, uncontrollable diarrhea. Labs drawn indicated hypokalemia, sustained neutropenia, and worsening thrombocytopenia. All stool studies (ova and parasites and stool cultures) and GI pathogen polymerase chain reaction (PCR) panels including *Clostridium difficile*, *shigella*, and *salmonella* were negative. He was started on filgrastim, cefepime, doxycycline, and vancomycin and placed on neutropenic precautions. The following day, he presented with worsening fever and thrombocytopenia. Atovaquone was initiated for *pneumocystis jirovecii* prophylaxis due to his low CD4+ count.

On day five, the patient continued to complain of severe pain, diffuse non-bloody diarrhea, significant hiccupping, and shortness of breath. Sputum cultures were unable to be obtained due to difficulty in obtaining samples. The patient appeared to have a candidal infection on his tongue; however, due to his macroglossia and xerostomia, further clinical examination was difficult to perform. He was started on micafungin. At this time, the patient was hypotensive and tachycardic with a blood pressure (BP) of 82/53 mmHg and heart rate (HR) of 108 beats per minute (bpm). Laboratory exams revealed continuing thrombocytopenia and neutropenia, RBCs of 2.96 g/dL, and critical anemia (Hgb 6.1 g/dL). Due to these findings, the patient received transfusions of both RBCs and platelets, which led to a temporary improvement in the labs. Preliminary blood cultures were positive for *E. coli* bacteremia, prompting a switch from cefepime and doxycycline to Meropenem. 

Despite the initiation of multiple antibiotics and transfusions, the patient continued to spike fevers throughout the night. The following day, the patient had fluctuating vitals with tachycardia, hypotension, and a fever of 102.7 °F. The patient was scheduled to remove his internal jugular (IJ) venous port as this may have been a nidus for his current infection. However, the procedure resulted in a rapid response with new-onset atrial fibrillation with a rapid ventricular response. He was then transferred to the intensive care unit due to decompensation with worsening of sepsis. He was sedated on dexmedetomidine and rate-controlled on amiodarone. He received several platelet and RBC transfusions.

**Figure 1:**
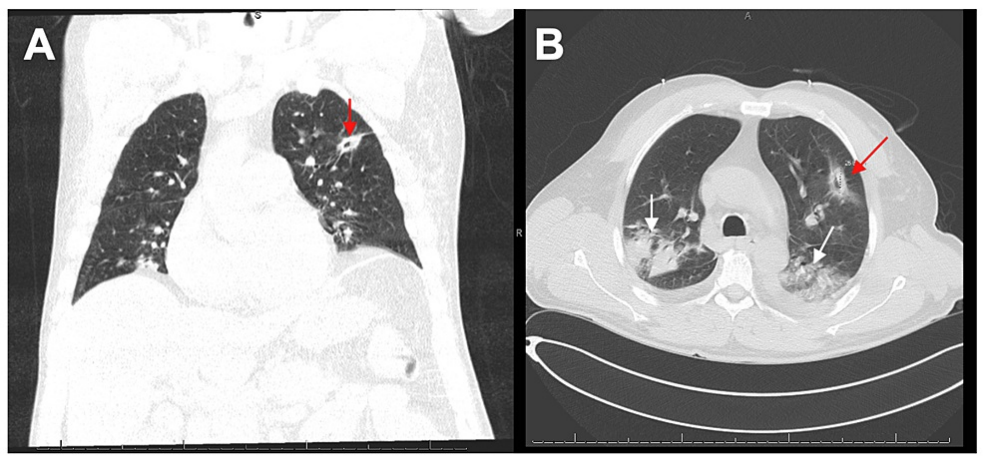
CT Chest without Contrast (A, B) Large multifocal airspace consolidations in the bilateral upper and lower lobes (white arrows). Pulmonary nodule with a central cavitary lesion seen on the left upper lobe (red arrows). Development of right upper lobe and bilateral lower lobe pneumonia.

Three days later, our patient stabilized and returned to the floor with continued complaints of shortness of breath, diarrhea, and now, worsening hiccups. He also had new complaints of dizziness, hoarseness, throat pain, and nosebleeds. On day eleven of his stay, he continued to have fevers and was noted to be in sinus tachycardia with oxygen desaturations throughout the night. Fungal cultures returned negative. A follow-up CT of the chest without contrast demonstrated extensive pulmonary disease, a left upper quadrant nodule with central cavitation, and development of right upper quadrant and bilateral lower lobe pneumonia (Figure 1). These findings were concerning for either pneumonia, cavitary abscess, or a metastatic lesion. At this time, the patient’s labs showed an absolute neutrophil count (ANC) < 3000 (normal: 5000/μL). The patient was started on ceftriaxone and amphotericin B due to suspicions of invasive aspergillosis infection. 

On day 14, the patient was found to have possible herpetic lesions that appeared on his lips and was empirically started on acyclovir. Lab studies for *histoplasmosis*, *cryptococcus,* and *aspergillus* galactomannan antigens returned negative. Clinical suspicion for HSV or CMV was raised, prompting PCR serology. Less likely differentials such as varicella zoster and impetigo were also considered. He continued to have elevated temperatures overnight, and amphotericin B was discontinued due to suspicions of fevers related to its administration. The following day, pharyngeal lesions were suspected to be seen on physical exam. The patient’s repeat CD4 count was found to be 13 cells/mm^3^. Stool studies returned negative for fecal occult blood, and iron and ferritin levels were within range. At this time, the patient was maintained on acyclovir. On day sixteen, the patient then began complaining of a substernal stabbing pain that was worse with eating. Due to these new complaints, esophagogastroduodenoscopy, colonoscopy, and bronchoscopy were also ordered. 

**Figure 2:**
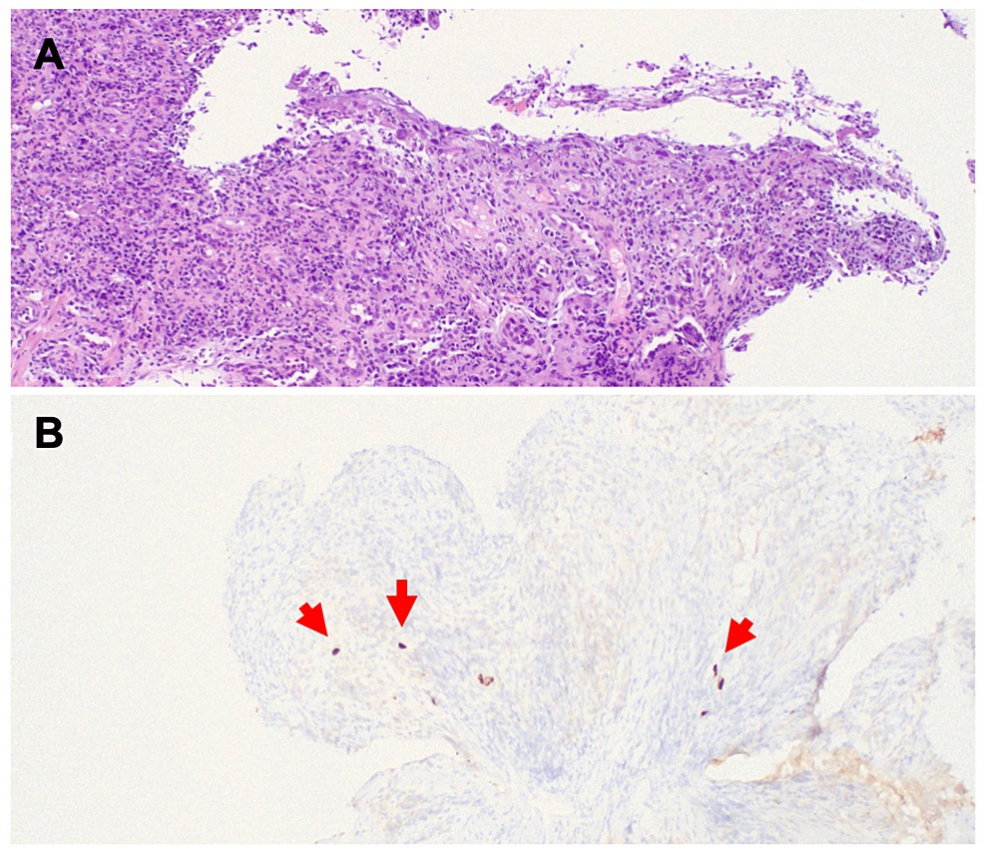
Histopathology of esophageal biopsy (A) H&E at 100x demonstrates esophagitis. Squamous epithelium is mostly replaced by a mixed inflammatory infiltrate. Occasional large nuclei can be seen in the upper aspect. (B) CMV immunostaining is positive in scattered cells (red arrows).

This further workup revealed a positive CMV viral load of 1374. Esophagogastroduodenoscopy revealed ulcerations with irregular margins and a yellowish-white base in the posterior pharyngeal area and on the vocal cords (laryngeal ulcers). Similar ulcers were also noted in the esophagus. No strictures were noted; however, a white exudate was seen throughout the esophagus. Gastritis was also noted in the stomach. Biopsies taken during the EGD were positively immunostained for CMV (Figure 2). Colonoscopy and bronchoscopy biopsies were negative at that time. He began ganciclovir 450 mg IV q12hr for fourteen days. Within this time, the patient's clinical status began to improve. He was then transitioned to oral valganciclovir 900 mg qd for six weeks upon discharge. During his stay, he was maintained on his home medications including ART. On discharge, he was instructed to continue atovaquone for PJP prophylaxis. He was recommended to follow up with ophthalmology to monitor for CMV retinitis as well as his primary care physician. 

## Discussion

This complicated case highlights the necessity to maintain a high index of suspicion for CMV EOD in immunocompromised patients presenting with confounding clinical symptoms and multiple comorbidities. CMV can have a slow onset and poor prognosis, requiring clinical diligence to prevent misdiagnosis and inadequate treatment. In our patient, chart reviews revealed a CD4 cell count of greater than 500 cells/mm^3^ and a well-controlled, undetectable viral load just one month prior to admission. Two weeks prior, CD4 counts were 212 cells/mm^3^. On the day of admission, CD4 counts were 42 cells/mm^3^. Two weeks into his hospital stay and near the time of CMV diagnosis, his CD4 was continuing to decline at 13 cells/mm^_3_^. This decline was most likely a side effect of his chemotherapy and radiation treatments, although it may be suggested that the additional insults of *E. coli* bacteremia and active CMV infection also played a role. 

CMV infections in immunocompetent hosts are either asymptomatic or may present as a self-limiting, mild mononucleosis-like sickness. Clinical features of CMV infections in immunocompromised patients may include fever, pharyngitis, pneumonia, hepatitis, encephalitis, myelitis, colitis, uveitis, retinitis, and neuropathy [1]. Additionally, patients undergoing treatment with immunosuppressive therapy or chemotherapy are at an increased risk for CMV infection and EOD [10]. Laboratory studies may reveal anemia, lymphocytosis with more than 10% atypical lymphocytes, abnormal liver function tests, thrombocytopenia or pancytopenia, positive rheumatoid factor, and positive antinuclear antibody levels [1]. Our patient falls under two of these categories of immunocompromised patients: infection with HIV and undergoing chemotherapy and radiation treatment, putting him at substantial risk for developing manifestations of CMV EOD. On his initial labs, he presented with pancytopenia, hyponatremia, thrombocytopenia, and AKI, although several of these labs are more likely due to his confounding conditions at presentation.

CMV esophagitis and colitis can present with odynophagia, abdominal pain, and bloody diarrhea. Endoscopic exam of the GI tract may show linear ulcers located in the middle to distal esophagus [8]. As mentioned, organ involvement in immunocompetent hosts is rare. However, CMV is an opportunistic pathogen and in patients with a newly suppressed immune system, as seen in our patient following chemotherapy sessions to treat his rectal squamous cell cancer, this serves as a perfect host for reactivation of or a new infection of CMV [1].

The gold standard for detecting CMV is histopathology identification of CMV inclusion bodies, commonly described as an “Owl’s eye” appearance [1,8]. However, active disease can also be detected by serology studies indicating IgM antibodies against CMV, which represents a new-onset infection, while a four-fold increase in IgG antibodies represents reactivation of a latent infection. However, immunosuppressed patients may have unreliable serological tests, so direct evidence of viremia may be helpful in ruling in the disease. This can include PCR testing to detect CMV DNA in the patient’s bodily fluids or an indirect immunofluorescence assay to detect CMV antigens [1,9].

It is important to note the slow progression of CMV in our patient. CMV expresses genes in a temporal manner, generally following a new onset of immunocompromised state [1]. Our patient underwent endoscopy with biopsies for severe gastroesophageal reflux a few weeks prior to admission, which only revealed gastritis and was negative for CMV. Therefore, at the time of admission, CMV ranked lower on the differential diagnosis. Additionally, it could be argued that our patient was not even affected by CMV at the time of admission. This presents an interesting question of the actual timing of CMV reactivation and the length of time it takes this virus to replicate and spread to its target tissues. Throughout his stay, our patient had worsening symptoms of pneumonia and severe diarrhea. This may be due to his recent chemotherapy sessions, or it may be other manifestations of CMV EOD. Biopsies were taken via bronchoscopy and colonoscopy to test for this, but both were negative. However, this does not definitively rule out the possibility of CMV EOD in these locations. Several factors need to be considered, including the chance that the virus was simply not biopsied at that time [11].

The prevention of CMV is best achieved with the use of ART to maintain CD4+ counts above 100 cells/mm^3^ [3]. This maintenance becomes complicated when patients are required to undergo other immunosuppressive treatments such as chemotherapy and radiation. Clinical guidelines no longer recommend CMV prophylaxis, which emphasizes the need for early CMV monitoring with appropriate physical examinations and symptom evaluation.

Our patient’s clinical course consisted of *E. coli* septicemia, new-onset atrial fibrillation with rapid ventricular response, worsening pneumonia, possible metastasis, progressive diarrhea, and potential oropharyngeal candidiasis. Despite broad-spectrum antimicrobial regimens, he remained symptomatic with new complaints of dysphagia and odynophagia. This complicated case highlights the necessity to maintain a high index of suspicion for rarer manifestations of CMV EOD, especially in the face of a multitude of pertinent clinical symptoms that may add to the differential diagnoses and delay appropriate targeted treatment. Inappropriately working-up and treating these patients, antimicrobial overuse may also be avoided and prevent the development of resistance. 

## Conclusions

This case highlights the possibility of CMV end-organ manifestations, even in the setting of a previously well-controlled HIV-positive patient on effective ART therapy. Our patient had a rapid development of multiple end-organ signs, which accentuates morbidity and mortality associated with CMV disease in the immunosuppressed state. This demonstrates how important it is to be aware of the multitude of opportunistic infections patients with HIV are susceptible to. Clinicians must maintain a high degree of suspicion for CMV and its EOD manifestations to avoid delays in diagnosis and worsening clinical outcomes. 
